# Multifunctional metasurfaces enabled by simultaneous and independent control of phase and amplitude for orthogonal polarization states

**DOI:** 10.1038/s41377-021-00552-3

**Published:** 2021-05-25

**Authors:** Mingze Liu, Wenqi Zhu, Pengcheng Huo, Lei Feng, Maowen Song, Cheng Zhang, Lu Chen, Henri J. Lezec, Yanqing Lu, Amit Agrawal, Ting Xu

**Affiliations:** 1 National Laboratory of Solid-State Microstructures, Jiangsu Key Laboratory of Artificial Functional Materials, College of Engineering and Applied Sciences, Nanjing University, 210093 Nanjing, China; 2 Collaborative Innovation Center of Advanced Microstructures, 210093 Nanjing, China; 3 Physical Measurement Laboratory, National Institute of Standards and Technology, Gaithersburg, MD 20877 USA; 4 Maryland NanoCenter, University of Maryland, College Park, MD 20877 USA; 5 School of Optical and Electronic Information and Wuhan National Laboratory for Optoelectronics, Huazhong University of Science and Technology, 430074 Wuhan, China

**Keywords:** Metamaterials, Sub-wavelength optics

## Abstract

Monochromatic light can be characterized by its three fundamental properties: amplitude, phase, and polarization. In this work, we propose a versatile, transmission-mode all-dielectric metasurface platform that can independently manipulate the phase and amplitude for two orthogonal states of polarization in the visible frequency range. For proof-of-concept experimental demonstration, various single-layer metasurfaces composed of subwavelength-spaced titanium-dioxide nanopillars are designed, fabricated, and characterized to exhibit the ability of polarization-switchable multidimensional light-field manipulation, including polarization-switchable grayscale nanoprinting, nonuniform cylindrical lensing, and complex-amplitude holography. We envision the metasurface platform demonstrated here to open new possibilities toward creating compact multifunctional optical devices for applications in polarization optics, information encoding, optical data storage, and security.

## Introduction

A coherent beam of monochromatic light can be characterized by three fundamental properties: phase, amplitude, and polarization^1^. For hundreds of years, humans have developed innovative approaches to manipulate these fundamental properties, resulting in bulk optical components omnipresent in current day technology, such as cellphones and cameras, as well as in complex free-space optical experiments, such as in the Laser Interferometer Gravitational-Wave Observatory (LIGO)^2^ or the recently demonstrated photonic quantum computer^3^. Typical examples of bulk optical components include optical lenses and spatial light modulators for phase control, neutral density filters for amplitude control, and waveplates for polarization control. In recent years, optical metasurfaces, which consist of an array of two-dimensional (2D) subwavelength nanostructures, has been shown to provide a compact and efficient platform for modifying the amplitude, phase, and polarization of light^4–14^. Various planar metasurface devices have been demonstrated to exhibit equivalent optical functionalities as their bulk counterparts, but instead in a significantly smaller and complementary metal oxide semiconductor-manufacturable footprint^15–23^. For example, metalenses—one typical subcategory of metasurface optics exhibiting only phase control—can offer desired phase engineering to achieve excellent diffraction-limited achromatic focusing for high-resolution imaging applications^24–31^.

Most common metasurface embodiments, such as a metalens, rely on nanostructure design targeted to manipulate only one property of light. While, in fact, each subwavelength nanostructure that constitutes an optical metasurface can be designed to instead simultaneously manipulate multiple properties of light. Therefore, it is possible to design single-layer meta-optic devices offering fascinating multifunctional responses and able to synchronously manipulate two or more properties of incident light, something that is either not possible with conventional optics, or require complex optical setups made up of two or more optical components. As an example, by simultaneously controlling the amplitude and phase of light at a desired wavelength, complex-amplitude holograms or synchronous nanoprinting holograms has been achieved using a single-layer metasurface-based optic^32–37^. Furthermore, various recently reported research results have demonstrated polarization-controlled phase-tuning metasurface optics to achieve polarization-switchable imaging^38–40^, holography^41–45^, and beam shaping^46,47^. However, how to interlink phase and amplitude with polarization to achieve encoding independent amplitude and phase functions into a pair of orthogonal polarization states of light is still a challenge. Such a capability can lead to the development of complex functionality, multichannel, and multifunctional meta-optical devices for engineering of light in a compact footprint. A plasmonic metasurface embodiment to realize independent amplitude and phase control of light for linearly polarization was recently demonstrated^48,49^. Nonetheless, this approach suffers from large losses due to ohmic absorption in the metal at optical frequencies, and only operated in refection mode, which significantly limits its applicability in common experimental scenarios.

In this work, we demonstrate a transmission-mode all-dielectric metasurface platform that can simultaneously and independently manipulate the amplitude and phase for a pair of orthogonal states of polarization at visible frequencies. In contrast to a previous demonstration that only relies on geometric-phase modulation to tune the amplitude and phase for a fixed polarization state^29^, here, by combining geometric phase with propagation phase, the proposed metasurface optics is able to completely decouple any combination of two arbitrary amplitude and phase profiles, and encode their information into two orthogonal polarization states. Various single-layer metasurfaces composed of subwavelength-spaced titanium-dioxide (TiO_2_) nanopillars on a fused-silica substrate are designed and fabricated to exhibit the ability of polarization-switchable multidimensional light-field manipulation. Examples of proof-of-concept experimental demonstrations shown here include polarization-switchable nanoprinting, nonuniform cylindrical lensing, and complex-amplitude holography. To the best of our knowledge, this is the first experimental realization of a single-layer metasurface that integrates four independent channels of different optical information for a pair of orthogonal polarization states. This capability is elegantly demonstrated here by incorporating two near-field nanoprinting images and two far-field hologram images, within the same metasurface. We envision this type of metasurface platform to open new possibilities of creating compact multifunctional optical devices for applications in polarization optics, information encoding, optical data storage, and security.

## Results

### Design principle

**Fig. 1:**
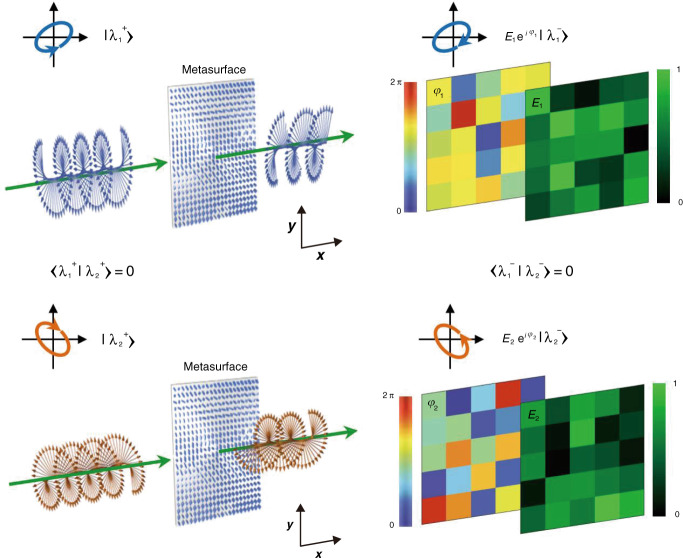
Conceptual illustration of a multifunctional metasurface. The proposed single-layer multifunctional metasurface can achieve arbitrary and independent amplitude and phase control for a pair of orthogonal polarization states. Upon transmission, the handedness of each output polarization (|*λ*_1_^−^〉, |*λ*_2_^−^〉) is flipped, with respect to the incident polarization (|*λ*_1_^+^〉, |*λ*_2_^+^〉). The output wavefront can possess any combination of arbitrary complex-amplitude distributions of $E_1e^{i\varphi _1}$ and $E_2e^{i\varphi _2}$. The phase and amplitude profiles (*φ*_1_, *φ*_2_, *E*_1_, and *E*_2_, respectively) for the two orthogonal polarization states are independent of each other

By leveraging the effective optical response of a metasurface to the three properties of incident light, a transmission-mode metasurface is envisioned to simultaneously and independently tailor the amplitude and phase for a pair of arbitrary orthogonal polarization states (this transformation is schematically shown in Fig. 1). When a monochromatic light beam with a specific state of input polarization |*λ*_1_^+^〉 is incident on the metasurface, the output wavefront can be described by an amplitude and phase distribution profiles of *E*_1_ and *φ*_1_, respectively. Similarly, when the input light beam is in the orthogonal state of polarization |*λ*_2_^+^〉 with respect to |*λ*_1_^+^〉 and is incident on the same metasurface, the output wavefront is described by two independent amplitude *E*_2_ and phase *φ*_2_ profiles. For a metasurface composed of linearly birefringent elements, there is also a restriction that the handedness of the output polarization is opposite to that of the input polarization. Therefore, for any two arbitrary orthogonal states of polarization, including circular, elliptical, or linear, the output wavefront has opposite-handedness polarization (|*λ*_1_^−^〉, |*λ*_2_^−^〉), with respect to the input polarization (|*λ*_1_^+^〉, |*λ*_2_^+^〉).

First, we consider two arbitrary orthogonal states of polarization in the linear polarization basis, |*λ*_1_^+^〉 = [cos *χe*^*iδ*^sin *χ*]^*T*^ and |*λ*_2_^+^〉 = [−sin *χe*^*iδ*^cos *χ*]^*T*^ incident on the metasurface, where parameter *χ* and *δ* determine the respective polarization states. As mentioned earlier, the output wavefronts are in the orthogonal polarization states, denoted by (|*λ*_1_^−^〉, |*λ*_2_^−^〉), and are complex conjugates of (|*λ*_1_^+^〉, |*λ*_2_^+^〉) with amplitude and phase profiles given by (*E*_1_, *E*_2_) and (*φ*_1_, *φ*_2_), respectively. The metasurface can be described by a Jones matrix *J*(*x*,*y*), which simultaneously satisfies the transformations, $J(x,y)\left| {\lambda _1^ + } \right\rangle = E_1(x,y)e^{i\varphi _1(x,y)}\left| {\lambda _1^ - } \right\rangle$ and $J(x,y)\left| {\lambda _2^ + } \right\rangle = E_2(x,y)e^{i\varphi _2(x,y)}\left| {\lambda _2^ - } \right\rangle$, at each spatial pixel location (*x*,*y*). The required Jones matrix is calculated as (see Supplementary Note 1 for details):

1 $$J(x,y) = \left[ {\begin{array}{*{20}{c}} {E_1\left( {x,y} \right)e^{i\varphi _1\left( {x,y} \right)}\cos ^2\chi + E_2\left( {x,y} \right)e^{i\varphi _2\left( {x,y} \right)}\sin ^2\chi } & {e^{ - i\delta }\cos \chi \sin \chi \left[ {E_1\left( {x,y} \right)e^{i\varphi _1\left( {x,y} \right)} - E_2\left( {x,y} \right)e^{i\varphi _2\left( {x,y} \right)}} \right]} \\ {e^{ - i\delta }\cos \chi \sin \chi \left[ {E_1\left( {x,y} \right)e^{i\varphi _1\left( {x,y} \right)} - E_2\left( {x,y} \right)e^{i\varphi _2\left( {x,y} \right)}} \right.} & {e^{ - i2\delta }\left[ {E_1\left( {x,y} \right)e^{i\varphi _1\left( {x,y} \right)}\sin ^2\chi + E_2\left( {x,y} \right)e^{i\varphi _2\left( {x,y} \right)}\cos ^2\chi } \right]} \end{array}} \right]$$

This is a non-unitary matrix and thus cannot be directly diagonalized. Mathematically, the complex amplitude $E_{1,2}\left( {x,y} \right)e^{i\varphi _{1,2}\left( {x,y} \right)}$ can be decomposed as:

2 $$E_{1,2}\left( {x,y} \right)e^{i\varphi _{1,2}\left( {x,y} \right)} = \cos \left( {\frac{{\varphi _{A}^{ + , - } - \varphi _B^{ + , - }}}{2}} \right)e^{i\left( {\varphi _A^{ + , - } + \varphi _B^{ + , - }} \right)/2} = \left( {e^{i\varphi _A^{ + , - }} + e^{i\varphi _B^{ + , - }}} \right)/2$$

where $E_{1,2}\left( {x,y} \right) = \cos \left( {\frac{{\varphi _A^{ + , - } - \varphi _B^{ + , - }}}{2}} \right)$ and $\varphi _{1,2}\left( {x,y} \right) = \left( {\varphi _A^{ + , - } + \varphi _B^{ + , - }} \right)/2$. By substituting Eq. (2) into Eq. (1), *J*(*x*,*y*) can be rewritten as:

3 $$J\left( {x,y} \right) = \left[ {J_A\left( {x,y} \right) + J_B\left( {x,y} \right)} \right]/2$$

where

4 $$J_A\left( {x,y} \right) = \left[ {\begin{array}{*{20}{c}} {e^{i\varphi _A^ + (x,y)}\cos ^2\,\chi + e^{i\varphi _A^ - (x,y)}\sin ^2\,\chi } & {e^{ - i\delta }\cos\, \chi \sin\, \chi \left[ {e^{i\varphi _A^ + \left( {x,y} \right)} - e^{i\varphi _A^ - \left( {x,y} \right)}} \right]} \\ {e^{ - i\delta }\cos\, \chi \sin\, \chi \left[ {e^{i\varphi _A^ + \left( {x,y} \right)} - e^{i\varphi _A^ - \left( {x,y} \right)}} \right]} & {e^{ - i2\delta }\left[ {e^{i\varphi _A^ + \left( {x,y} \right)}\sin ^2\,\chi + e^{i\varphi _A^ - \left( {x,y} \right)}\cos ^2\,\chi } \right]} \end{array}} \right]$$

and

5 $$J_B\left( {x,y} \right) = \left[ {\begin{array}{*{20}{c}} {e^{i\varphi _B^ + (x,y)}\cos ^2\,\chi + e^{i\varphi _B^ - (x,y)}\sin ^2\,\chi } & {e^{ - i\delta }\cos\, \chi \sin\, \chi \left[ {e^{i\varphi _B^ + \left( {x,y} \right)} - e^{i\varphi _B^ - \left( {x,y} \right)}} \right]} \\ {e^{ - i\delta }\cos\, \chi \sin\, \chi \left[ {e^{i\varphi _B^ + \left( {x,y} \right)} - e^{i\varphi _B^ - \left( {x,y} \right)}} \right]} & {e^{ - i2\delta }\left[ {e^{i\varphi _B^ + \left( {x,y} \right)}\sin ^2\,\chi + e^{i\varphi _B^ - \left( {x,y} \right)}\cos ^2\,\chi } \right]} \end{array}} \right]$$

In terms of the unitary conditions, *J*_*A*_(*x*,*y*) and *J*_*B*_(*x*,*y*) can be diagonalized by solving their characteristic equations and rewritten in a standard form *J*_*A*_(*x*,*y*) = *R*_*A*_*Λ*_*A*_*R*_*A*_^−1^ and *J*_*B*_(*x*,*y*) = *R*_*B*_*Λ*_*B*_*R*_*B*_^−1^, respectively, where *R*_*A,B*_ is a real unitary matrix and *Λ*_*A,B*_ is a diagonal matrix. This new formula for *J* (*x*,*y*) indicates that a metasurface composed of two different types of linearly birefringent optical elements can achieve the aforementioned transformations. The eigenvectors and eigenvalues of *J*_*A*_(*x*,*y*) and *J*_*B*_(*x*,*y*) determine the required fast axis orientation angles and phase shifts of the two linearly birefringent optical elements, respectively.

Next, we describe the metasurface design procedure to achieve arbitrary and independent amplitude (*E*_1_, *E*_2_) and phase (*φ*_1_, *φ*_2_) control for any two orthogonal states of input polarization. First, for two orthogonal circular polarizations (*χ* = *π*/4 and *δ* = *π*/2), the analytical solutions for the required phase shifts and orientation angles of nanopillar A and nanopillar B are calculated as (see Supplementary Note. 2 for details)

Nanopillar A:

6 $$\left. {\begin{array}{*{20}{c}} {\delta _{Ax} = [{\mathrm{cos}}^{ - 1}E_1\left( {x,y} \right) + \varphi _1\left( {x,y} \right) + {\mathrm{cos}}^{ - 1}E_2\left( {x,y} \right) + \varphi _2\left( {x,y} \right)]/2} \\ {\delta _{Ay} = [{\mathrm{cos}}^{ - 1}E_1\left( {x,y} \right) + \varphi _1\left( {x,y} \right) + {\mathrm{cos}}^{ - 1}E_2\left( {x,y} \right) + \varphi _2\left( {x,y} \right)]/2 - \pi } \\ {\theta _A = [\varphi _1\left( {x,y} \right) + {\mathrm{cos}}^{ - 1}E_1\left( {x,y} \right) - \varphi _2\left( {x,y} \right) - {\mathrm{cos}}^{ - 1}E_2\left( {x,y} \right)]/4} \end{array}} \right\}$$

Nanopillar B:

7 $$\left. {\begin{array}{*{20}{c}} {\delta _{Bx} = [\varphi _1(x,y) - {\mathrm{cos}}^{ - 1}E_1(x,y) + \varphi _2(x,y) - {\mathrm{cos}}^{ - 1}E_2\left( {x,y} \right)]/2} \\ {\delta _{By} = [\varphi _1(x,y) - {\mathrm{cos}}^{ - 1}E_1(x,y) + \varphi _2(x,y) - {\mathrm{cos}}^{ - 1}E_2\left( {x,y} \right)]/2--\pi } \\ {\theta _B = [\varphi _1(x,y) - {\mathrm{cos}}^{ - 1}E_1(x,y) - \varphi _2(x,y) + {\mathrm{cos}}^{ - 1}E_2\left( {x,y} \right)]/4} \end{array}} \right\}$$

Note that for input orthogonal circular polarization states, a combination of geometric phase and propagation phase is required to achieve independent amplitude and phase control. These equations determine the size and orientation of the nanopillars at any spatial coordinates (*x*,*y*) of the metasurface.

Second, for two orthogonal linear polarizations (*χ* = 0 and *δ* = 0), we can also obtain analytical solution of Jones matrix in the linear polarization basis. The required phase shifts of nanopillars are expressed as (see Supplementary Note. 2 for details):

Nanopillar A:

8 $$\left. {\begin{array}{*{20}{c}} {\delta _{Ax} = \cos ^{ - 1}E_1\left( {x,y} \right) + \varphi _1\left( {x,y} \right)} \\ {\delta _{Ay} = \cos ^{ - 1}E_2\left( {x,y} \right) + \varphi _2\left( {x,y} \right)} \end{array}} \right\}$$

Nanopillar B:

9 $$\left. {\begin{array}{*{20}{c}} {\delta _{Bx} = \varphi _1\left( {x,y} \right) - \cos ^{ - 1}E_1\left( {x,y} \right)} \\ {\delta _{By} = \varphi _2\left( {x,y} \right) - \cos ^{ - 1}E_2\left( {x,y} \right)} \end{array}} \right\}$$

For orthogonal linear polarization states, only propagation phase is required to achieve the desired amplitude and phase control. Equations (6)-(9) determine the analytical solutions for control of orthogonal circular and linear polarization states. For a more general case of elliptical polarization, although the eigenvalues and eigenvectors of *J*_*A*_ (*x*,*y*) and *J*_*B*_ (*x*,*y*) do not yeild analytical solutions for the required phase shifts and orientation angles of nanopillar A and nanopillar B, numerical solutions can instead be employed to achieve the requisite nanopillar design for a desired metasurface response.

**Fig. 2:**
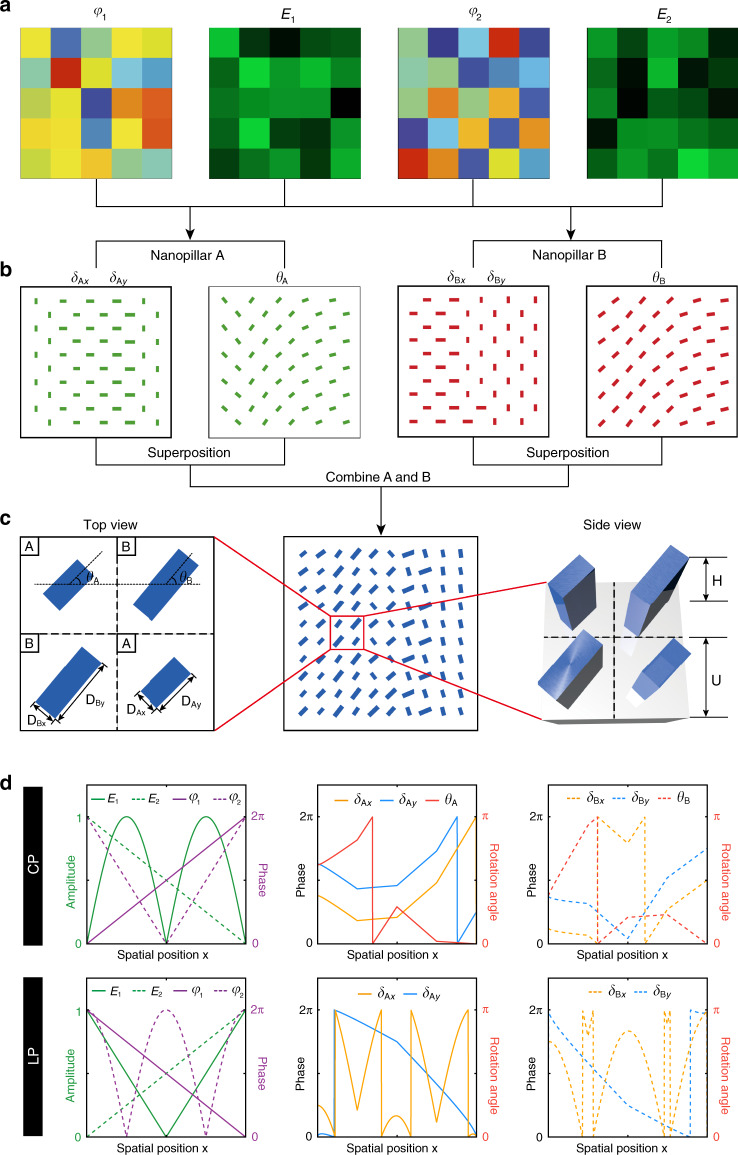
Design principle of a multifunctional metasurface. **a**–**c** A general design flow of multifunctional metasurface for independent phase and amplitude control of orthogonal polarization states. **a** The target amplitude and phase profiles encoded on the metasurface. **b** The dimensions and orientation angles of nanopillars A and B are determined by the calculation results of *δ*_*Ax*_, *δ*_*Ay*_, *θ*_*A*_ *δ*_*Bx*_, *δ*_*By*_, and *θ*_*B*_. **c** The fabricated multifunctional metasurface is composed of TiO_2_ rectangular nanopillars with same height *H*. One superpixel of the metasurface consists of four nanostructures, two nanopillar A and two nanopillar B, which are alternately arranged on a fused-silica substrate in a square lattice *U*. **d** Top panel: for orthogonal circular polarizations and given arbitrary amplitude and phase distribution of *E*_1_ = |sin *x*|, *E*_2_ = 1 − *x*/2*π*, *φ*_1_ = *x* and *φ*_2_ = |2*π* − 2*x|* (left), where *x* ∈ [0, 2*π*], the required phase shifts and orientation angles of the nanopillar A (middle) and nanopillar B (right) can be obtained. Bottom panel: for orthogonal linear polarizations and given arbitrary amplitude and phase distribution of *E*_1_ = *x*/*π* − 1, *E*_2_ = *x*/2*π*, *φ*_1_ = |*x* − 2*π*| and *φ*_2_ = |2*π* cos *x*| (left), where *x* ∈ [0, 2*π*], the required phase shifts of the nanopillar A (middle) and nanopillar B (right) can be obtained, respectively.

The design flow of multifunctional metasurface is illustrated in Fig. 2a–c. First, according to the desired functionality of the required metasurface optics, the target complex-amplitude profiles ($E_1e^{i\varphi _1}$ and $E_2e^{i\varphi _2}$) encoded on the metasurface are determined (Fig. 2a). Second, given a pair of orthogonal circular or linear polarization states, the required phase shifts (*δ*_*Ax*_, *δ*_*Ay*_, *δ*_*Bx*_, and *δ*_*By*_) and orientation angles (*θ*_*A*_ and *θ*_*B*_) provided by nanopillar A and nanopillar B can be calculated based on Eqs. (6)–(9), respectively. Then the dimensions and rotation angles of the mapped nanopillars A and B are determined by the calculated phase shifts and orientation angles (Fig. 2b). Finally, by combining nanopillar A with nanopillar B, the entire metasurface is successfully created (middle panel of Fig. 2c). Nanopillar A and nanopillar B, made of TiO_2_ are alternately arranged in a 2D square grid (with nominal lattice constant *U* = 450 nm) on a fused-silica substrate. Four TiO_2_ nanopillars arranged on a 2 × 2 square grid are used to define one metasurface superpixel. Each rectangular TiO_2_ nanopillar is chosen to be of a fixed nominal height *H* = 600 nm and their in-plane dimensions *D*_*Ax*_, *D*_*Ay*_, *D*_*Bx*_, and *D*_*By*_, respectively, determine the propagation phase shifts *δ*_*Ax*_, *δ*_*Ay*_, *δ*_*Bx*_, and *δ*_*By*_ along the nanopillars’ symmetry axes. The geometric phases are, respectively, controlled by the orientation angle *θ*_*A*_ and *θ*_*B*_ of the nanopillar relative to its fast axis. According to the desired phase shifts derived from the solution of the metasurface Jones matrix, a set of nanopillar satisfying the phase requirement while exhibiting a relatively high transmission efficiency is selected from the simulation library for the metasurface design.

Based on above design guidelines, Fig. 2d shows an example where the desired arbitrary and independent amplitude and phase distributions, (*E*_1_, *E*_2_) and (*φ*_1_, *φ*_2_), respectively, for any two orthogonal states of polarization are plotted. The corresponding phase shifts along the two symmetry axes and orientation angles of nanopillars are calculated and shown in the top panel of Fig. 2d for orthogonal circular polarization states, and bottom panel of Fig. 2d for orthogonal linear polarization states.

### Independent amplitude control of orthogonal states of input polarization

**Fig. 3:**
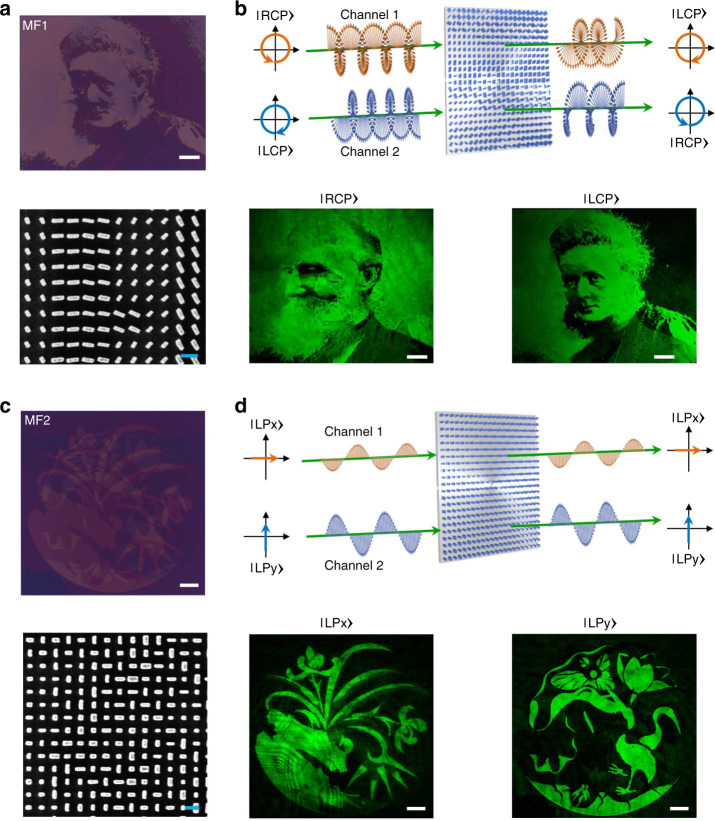
Experimental demonstration of two channel metasurface nanoprinting enabled by amplitude control of orthogonal polarization states. **a** Top: optical microscope image of the fabricated metasurface device MF1. Scale bar: 50 μm. Bottom: scanning electron microscopy (SEM) image of MF1. Scale bar: 500 nm. **b** Top: schematic illustration of device MF1 for switchable nanoprinting, using two orthogonal circular polarization channels. Bottom: experimental results for chirality-dependent nanoprinting grayscale portraits. By switching RCP light to LCP light, Kelvin’s portrait and Madame Curie’s portrait can be obtained in the near field, respectively. Scale bar: 50 μm. **c** Top: optical microscope image of the fabricated metasurface device MF2. Scale bar: 50 μm. Bottom: SEM image of metasurface MF2. Scale bar: 500 nm. **d** Top: schematic illustration of device MF2 for switchable nanoprinting, using two orthogonal linear polarization channels. Bottom: polarization-dependent metasurface nanoprinting generated plants images of “orchids” for *x*-polarized light and “lotuses” for *y*-polarized light. Scale bar: 50 μm

For simplicity, we first experimentally demonstrate the ability of the proposed metasurface platform to achieve independent amplitude control for circular and linear orthogonal states of input polarization. To illustrate this capability, we design a metasurface (labeled MF1) to imprint nanoprinting images of Kelvin’s portrait and Madame Curie’s portrait, respectively, to the two orthogonal circular polarization states of incident light. A second metasurface (labeled MF2) is designed to imprint nanoprinting images of “orchids” and “lotuses” to the two orthogonal linear polarization states. Based on the requisite intensities of the desired grayscale images, the lateral size and orientation of each nanopillar as a function of the spatial coordinates (*x*,*y*) in the metasurface plane are calculated (Figs. S1 and S2). The metasurface devices are fabricated using electron-beam (e-beam) lithography followed by atomic layer deposition (ALD) and dry etching process (see “Methods” section for details). Figure 3a shows the optical microscopy and scanning electron microscopy (SEM) images of the fabricated metasurface device (MF1). The fabricated sample is illuminated by a collimated beam, at the design wavelength of ~530 nm, generated from a semiconductor laser. The intensity distribution of the transmitted light after exiting the metasurface is captured using a 20× objective lens, and subsequently imaged with a charge-coupled device camera. Schematic of the experimental setup is shown in Fig. S3. By using a combination of linear polarizer and a quarter waveplate (QWP), light incident on MF1 is converted to the desired circular polarized state. By rotating the fast axis of QWP from 45° to −45°, we capture two independent grayscale nanoprinting images, Kelvin’s portrait for right-circular polarization (RCP) and Madame Curie’s portrait for the left-circular polarization (LCP), as shown in Fig. 3b. Because of the ability of the metasurface device to spatially control amplitude at will, it can be clearly seen that the metasurface-generated nanoprinting images for the two orthogonal input polarization states exhibit the desired response with high optical contrast and low crosstalk. Equivalently, Fig. 3c shows the optical microscopy and SEM images of the metasurface device MF2. By replacing QWP with a half waveplate (HWP) and rotating the fast axis of HWP from 0° to 45°, we capture, in transmission, two nanoprinting images, “orchids” for linearly polarized input light along the *x*-axis, and “lotuses” for linear polarization along the *y*-axis (Fig. 3d). Similar to MF1, the distinct spatial features on each of the acquired nanoprinting images can be easily distinguished with high contrast. The results presented in Fig. 3c, d clearly illustrate the ability of proposed platform to achieve independent amplitude control for two orthogonal states of polarization with high fidelity.

### Simultaneous and independent phase and amplitude control of orthogonal states of input polarization

**Fig. 4:**
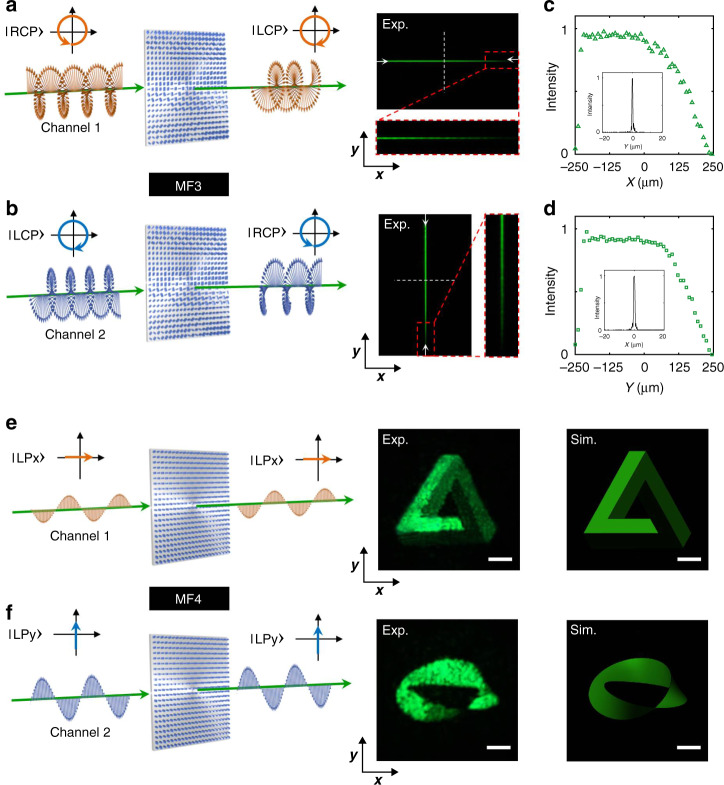
Multifunctional metasurface devices enabled by independent amplitude and phase control for orthogonal polarization states. **a**, **b** Experimental demonstration of chirality-dependent cylindrical lens line focusing with nonuniform intensity distribution. The device is designed to focus RCP and LCP light to a line along the *x*-direction (right of **a**) and *y*-direction (right of **b**), along with a brightness gradient, at a focal length of ~0.5 and 1.0 mm, respectively. **c**, **d** Cross-sections of the measured RCP and LCP line foci along the *x*- and the *y*-directions (denoted by two white arrows of **a** and **b**), respectively. Insets: intensity distributions of the middle of measured RCP and LCP line foci along *y*- and *x-*directions (denoted by dashed white lines of **a** and **b**). **e**, **f** Experimental demonstration of polarization-dependent complex-amplitude holograms. Right: experimental and calculated intensity distributions of far-field hologram images of “Penrose triangle” for *x*-polarized light (**e**) and “Mobius strip” for *y*-polarized light (**f**). Scale bar: 200 μm. Experimental images were obtained at a propagation distance of *z* ≈ 5 mm

In addition to only controlling amplitude or phase independently for the two orthogonal polarization states, the proposed metasurface platform can simultaneously and independently control both amplitude and phase for the two polarizations. As an experimental demonstration, we first design a metasurface device (labeled MF3) to realize polarization-dependent cylindrical lens focusing with nonuniform intensity distributions for two orthogonal circular polarization states (RCP and LCP). The corresponding cylindrical lens phase and amplitude profiles encoded on the metasurface for the two orthogonal circular polarization states are given in Fig. S4. The phase shifts (*δ*_*Ax*_, *δ*_*Ay*_, *δ*_*Bx*_, and *δ*_*By*_) and rotation angles (*θ*_*A*_ and *θ*_*B*_) of the nanopillars A and B as a function of spatial coordinates (*x*,*y*) in the plane of metasurface (MF3) are shown in Fig. S5. Upon illumination of the metasurface MF3 with RCP light, a line focus along the *x*-direction in the transverse *x*–*y* plane is captured approximately at the design focal length of *z* = 0.5 mm (Fig. 4a). By switching the input polarization to LCP, a focal line along the *y*-direction in the *x*–*y* plane is captured approximately at a different design focal length of *z* = 1.0 mm (Fig. 4b). The intensity cross-sections of the two-line focus along the *x*- and the *y*-directions are shown in Fig. 4c, d, respectively. The cylindrical lens function only utilized the ability of the metasurface to control independent phase profiles for the two polarizations; however, to illustrate simultaneous control of amplitude, the metasurface line focus was designed to linearly vary in intensity along its length. The superimposed simultaneous amplitude control results in the measured line focus intensity for RCP illumination to decrease gradually from left to right (along the *x*-direction), and for LCP illumination to decrease gradually from top to down (along the *y*-direction). As shown in the insets of Fig. 4c, d, the measured full-width at half-maximum of the line focus for RCP and LCP light are, respectively, 723 nm (±15 nm) and 1252 nm (±21 nm), which are close to the theoretical values of 612 and 1125 nm, as calculated by the Rayleigh criterion (0.514*λ*/NA, where NA is the numerical aperture of the cylindrical lens).

In addition to the chirality-dependent nonuniform cylindrical lensing function, we design another metasurface (labeled MF4) to achieve disparate complex-amplitude holograms for two orthogonal states of linear polarization. The complex-amplitude holograms generated by a set of independently controlled amplitude and phase functions are computed by the Fresnel diffraction formula. The near-field amplitude and phase profiles that can produce two independent far-field images of a “Penrose triangle” for *x*-polarized light, and a “Mobius strip” for *y*-polarized light, are shown in Fig. S6. The spatial distribution of the required phase shifts imparted by the nanopillars A and B along the metasurface plane are shown in Fig. S7. Experimentally, by changing the orientation of linear polarization incident on the metasurface MF4 from *x*-polarization to *y*-polarization, two holograms, a “Penrose triangle” (Fig. 4e) and a “Mobius strip” (Fig. 4f) are successfully captured at a distance of *z* ≈ 5 mm away from the metasurface-exit plane. The vivid three-dimensional visual effects with regions of bright and dark contrast in the captured hologram images are made possible by the ability to control amplitude and phase simultaneously in the proposed platform. The difference between the experimental and simulated results mainly arises from limited sampling of target amplitude and phase encoded on the metasurface and the morphological deviations (such as disparity of diameters, heights, or roughness) between the designed and the fabricated nanopillars. These experimental results, both for orthogonal circular and linear polarizations, clearly verify that metasurface platform can simultaneously and independently control both the phase and amplitude profiles for two orthogonal states of input polarization.

### Synchronous four-channel nanoprinting-hologram generation using a metasurface

**Fig. 5:**
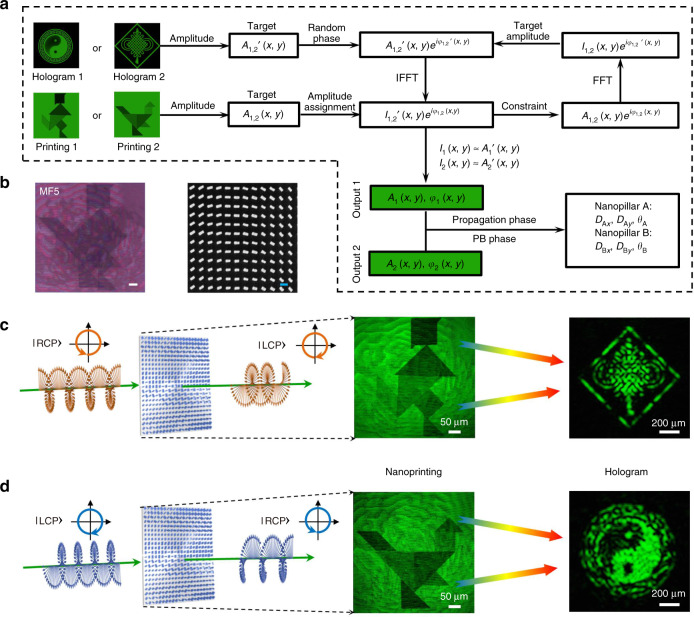
Synchronous generation of four-channel nanoprinting hologram using a metasurface device. **a** Design flow chart of a modified Gerchberg–Saxton algorithm for polarization-dependent synchronous nanoprinting-hologram generation using a metasurface. **b** Left: optical microscope image of the fabricated metasurface device MF5. Scale bar: 50 μm. Right: SEM image of MF5. Scale bar: 500 nm. **c**, **d** Experimental results of generation of four-channel nanoprinting-hologram images: nanoprinting images of “person” (RCP) and “bird” (LCP) near the metasurface, and hologram images of “Chinese knot” (RCP) and “Eight Trigram” (LCP) in the far field

Benefitting from the freedom in design and the multifunctional response of the proposed metasurface platform, we experimentally demonstrate, for the first time to our knowledge, imprinting of four arbitrary independent images, consisting of two near-field nanoprinting images and two far-field hologram images, all encoded onto a single-layer metasurface. Figure 5a illustrates the metasurface design flow chart for synchronous four-channel nanoprinting-hologram image generation based on a modified Gerchberg–Saxton algorithm^50^. First, we extract the amplitude of target hologram image in the far-field *A*_1,2_′(*x*,*y*) and add a random phase *φ*_1,2_′(*x*,*y*) to it. An inverse fast Fourier transform (FFT) step is implemented to the constructed complex amplitude. Second, the amplitude *I*_1,2_′(*x*,*y*) of generated complex amplitude is substituted with the near-field amplitude *A*_1,2_(*x*,*y*) of the nanoprinting image. Utilizing a FFT step to $A_{1,2}(x,y)e^{i\varphi _{1,2}(x,y)}$, the new complex amplitude at the target hologram plane can be obtained and its amplitude of *I*_1,2_(*x*,*y*) is replaced by the amplitude *A*_1,2_′(*x*,*y*) of the target hologram image. After few iterations, when the computed intensity *I*_1,2_(*x*,*y*) at the far-field hologram plane is close to the target amplitude *A*_1,2_′(*x*,*y*), we can obtain the phase distribution *φ*_1,2_(*x*,*y*) at the near-field nanoprinting plane. The same sequence of steps is then repeated for the other orthogonal state of polarization. Finally, by combining geometric phase and propagation phase modulation, the dimensions and orientations of nanopillars at each spatial point (*x*,*y*) on the metasurface plane can then be determined by the amplitude *A*_1,2_(*x*,*y*) and phase *φ*_1,2_(*x*,*y*) for the two orthogonal states of light.

For the proof-of-concept demonstration of this concept, we chose two orthogonal circular polarization input states to design the metasurface device for synchronous generation of four-channel nanoprinting-hologram images. The computed phases and amplitudes are shown in Fig. S8. Accordingly, the phase shifts and rotation angle of the nanopillars A and B as a function of spatial coordinates in the metasurface (labeled MF5) plane are shown in Fig. S9. The optical microscopy and SEM images of the fabricated metasurface MF5 are shown in Fig. 5b. For RCP incident light, a nanoprinting image of “person” is captured near the metasurface-exit surface, and a hologram image of “Chinese knot” is simultaneously captured at a propagation distance of *z* ≈ 5 mm away from the metasurface (Fig. 5c). For LCP incident light, a nanoprinting image of “bird” is captured near the metasurface and a hologram image of “Eight Trigram” is captured at *z* ≈ 5 mm (Fig. 5d). The measured nanoprinting images near the metasurface are composed of several blocks with varying brightness, and are consistent with the original images. The experimental results also agree well with the simulation predictions for the reconstructed far-field hologram images (Fig. S10).

## Discussion

In summary, we demonstrated a transmission-mode metasurface platform for simultaneous and independent control of phase and amplitude for two orthogonal states of input polarization. A single-layer dielectric metasurface device, composed of polarization-dependent birefringent nanopixels and leveraging both geometric and propagation phase modulations, is shown to directly achieve complex multidimensional wavefront transformations. As a proof-of-concept demonstration, we designed, fabricated, and characterized a series of metasurface devices, based on TiO_2_ as the constituent material, with polarization-switchable light-field manipulation capabilities, including near-field nanoprinting, far-field complex-amplitude holography, and nonuniform cylindrical focusing. Finally, benefiting from the design freedom of the proposed metasurface platform, a four-channel metasurface is experimentally demonstrated to realize the integration of four independent images for switchable synchronous nanoprinting and holography.

Due to its broadband response (Fig. S11), in principle, our proposed transmission dielectric metasurface platform can be designed to generate full-color holograph by leveraging sensitivity of higher order diffraction in metasurface to wavelength and angle^49^. Another alternative approach would be to design a metasurface with spatial multiplexed superpixels, where each superpixel consists of nanopillars that offer multilevel phases for each of the R, G, and B component, enabling realization of polarization-dependent full-color nanoprinting and holograph display. We envision this work to inspire creation of ultracompact flat-profile nanophotonic platforms, and provide new avenues for applications in polarization optics, information security, optical data storage, and multifunctional photonics.

## Materials and methods

### Numerical simulation of metasurface

Full-wave numerical simulations are performed using the finite-difference time-domain technique. Rectangular TiO_2_ nanopillars with a fixed height of 600 nm are arranged on a fused-silica substrate with a lattice constant of 450 nm. The complex refractive index of TiO_2_ as a function of wavelength is shown in Fig. S12. The incident plane wave is polarized along *x*- or *y*-axes, and illuminates the nanopillars from the substrate side. Along *x*- and *y*-axes, periodic boundary conditions are applied and perfectly matched layer boundary condition is used in the *z*-direction. The phase shifts (*P*_*x*_ and *P*_*y*_) and power transmission (*T*_*x*_ and *T*_*y*_) are obtained by parameter sweeping of the in-plane dimensions (*D*_*x*_ and *D*_*y*_) of the nanopillars by varying them between 50 and 350 nm at an interval of 5 nm (Fig. S13). As shown in Fig. S14, the optical fields are all confined within each nanopillar guaranteeing that a superpixel composed of four nanopillars can approximate a local pixel in the Jones matrix.

### Nanofabrication of metasurface devices

At first, a nominally double-side polished fused-silica substrate was vapor-coated with a monolayer of hexamethyldisilazane, and then a layer of ~600 nm thick positive-tone e-beam resist was spin-coated onto it. In order to suppress the charging effect during the e-beam lithography step, the sample was coated with a thin layer of aluminum via thermal evaporation. Afterward, e-beam lithography (at a nominal accelerating voltage of 100 kV and beam current of 2 nA) and resist development (in hexyl-acetate for ~120 s) was performed. Next, the patterned sample was coated with TiO_2_ using ALD at a low temperature of ~90 °C, and the overcoated TiO_2_ layer was etched using an inductively coupled plasma reactive-ion etching, with a gas mixture of Cl_2_ and BCl_3_. The etching was stopped when the overcoated TiO_2_ has been fully removed and the e-beam resist was exposed. Finally, after exposed to ultraviolet irradiation, the resist is removed by soaking in *n*-methyl-2-pyrrolidone and the array of TiO_2_ nanopillars is obtained.

## Supplementary information

SUPPLEMENTAL MATERIAL
