# Supplementary material for: Multifunctional metasurfaces enabled by simultaneous and independent control of phase and amplitude for orthogonal polarization states
Source: Light Sci Appl. 2021 May 25;10:107. doi: 10.1038/s41377-021-00552-3 (PMC8149653; doi:10.1038/s41377-021-00552-3)
Supplement: Supplementary file 1 — SUPPLEMENTAL MATERIAL [file 41377_2021_552_MOESM1_ESM.docx]

**Supplementary Information for “Multifunctional metasurfaces enabled by simultaneous and independent control of phase and amplitude for orthogonal polarization states”**

Mingze Liu^1,2^, Wenqi Zhu^3,4^, Pengcheng Huo^1,2^, Lei Feng^1,2^, Maowen Song^1,2^, Cheng Zhang^5^, Lu Chen^3,4^, Henri J. Lezec^3^, Yanqing Lu^1,2^, Amit Agrawal^3,4^ and Ting Xu^1,2^

1. *National Laboratory of Solid-State Microstructures, Jiangsu Key Laboratory of Artificial Functional Materials, College of Engineering and Applied Sciences, Nanjing University, Nanjing 210093, China*
2. *Collaborative Innovation Center of Advanced Microstructures, Nanjing 210093, China*
3. *Physical Measurement Laboratory, National Institute of Standards and Technology, Gaithersburg, MD 20877, USA*
4. *Maryland NanoCenter, University of Maryland, College Park, MD 20877, USA*
5. *School of Optical and Electronic Information and Wuhan National Laboratory for Optoelectronics, Huazhong University of Science and Technology, Wuhan 430074, China.*

**Supplementary Note 1. Derivation of the Jones matrix** $\boldsymbol{J(x,y)}$ **of the proposed metasurface:**

As mentioned in the main text, the proposed metasurface can encode two independent complex amplitude profiles on two input orthogonal polarization states, and the output states of polarization are complex conjugates of the input polarization. We first assume two arbitrary orthogonal states of polarization pair {$\left. |{\lambda_{1}}^{+} \right\rangle, \left. |{\lambda_{2}}^{+} \right\rangle$} in the linear polarization basis:

$\left. |{\lambda_{1}}^{+} \right\rangle={[\begin{matrix} \cos\chi& e^{i\delta}\sin\chi\end{matrix} ]}^{T}$ (S1)

$\left. |{\lambda_{2}}^{+} \right\rangle{=[\begin{matrix} -sin \chi& e^{i\delta}\cos\chi\end{matrix} ]}^{T}$ (S2)

where $\chi$ and *δ* set the elliptical polarization states. Each element constituting the metasurface can be described by a Jones matrix $J(x,y)$ and each pixel (*x*, *y*) on the metasurface should impart two uncorrelated complex amplitude $E_{1}(x,y)e^{i\varphi_{1}(x,y)}$ and $E_{2}(x,y)e^{i\varphi_{2}(x,y)}$. The two transformations carried out by the Jones matrix $J(x,y)$ are expressed by:

$\left. J(x,y)|{\lambda_{1}}^{+} \right\rangle=E_{1}(x,y)e^{i\varphi_{1}(x,y)}\left. {{|\lambda}_{1}}^{-} \right\rangle$ (S3)

$\left. J(x,y)|{\lambda_{2}}^{+} \right\rangle=E_{2}(x,y)e^{i\varphi_{2}(x,y)}\left. {{|\lambda}_{2}}^{-} \right\rangle$ (S4)

where the output polarization pairs {$\left. |{\lambda_{1}}^{-} \right\rangle, \left. |{\lambda_{2}}^{-} \right\rangle$} is the complex conjugate of the input polarization pair {$\left. |{\lambda_{1}}^{+} \right\rangle, \left. |{\lambda_{2}}^{+} \right\rangle$}. By substituting eq. S1 and eq. S2 into eq. S3 and eq. S4, matrix inversion of eq. S3 and eq. S4 results in:

$J \left( x, y \right)=\left[ \begin{matrix} E_{1}\left( x, y \right)e^{{i\varphi}_{1}\left( x, y \right)}{(cos \chi)}^{*} & E_{2}\left( x, y \right)e^{{i\varphi}_{2}\left( x, y \right)}{(-sin \chi)}^{*} \\ E_{1}\left( x, y \right)e^{{i\varphi}_{1}\left( x, y \right)}{(e^{i\delta}\sin\chi)}^{*} & E_{2}\left( x, y \right)e^{{i\varphi}_{2}\left( x, y \right)}{(e^{i\delta}\cos\chi)}^{*} \end{matrix} \right] \left[ \begin{matrix} \cos\chi& -sin \chi\\ e^{i\delta}\sin\chi& e^{i\delta}\cos\chi\end{matrix} \right]^{-1}$ (S5)

By carrying out the matrix multiplication, the required Jones matrix is calculated as:

$J \left( x, y \right)=\left[ \begin{matrix} E_{1}\left( x, y \right)e^{{i\varphi}_{1}\left( x, y \right)}\cos^{2} \chi+E_{2}\left( x, y \right)e^{{i\varphi}_{2}\left( x, y \right)}\sin^{2} \chi& e^{-i\delta}\cos\chi\sin\chi[E_{1}\left( x, y \right)e^{{i\varphi}_{1}\left( x, y \right)}-E_{2}\left( x, y \right)e^{{i\varphi}_{2}\left( x, y \right)}] \\ e^{-i\delta}\cos\chi\sin\chi[E_{1}\left( x, y \right)e^{{i\varphi}_{1}\left( x, y \right)}-E_{2}\left( x, y \right)e^{{i\varphi}_{2}\left( x, y \right)} & e^{-i2\delta}{[E}_{1}\left( x, y \right)e^{{i\varphi}_{1}\left( x, y \right)}\sin^{2} \chi+E_{2}\left( x, y \right)e^{{i\varphi}_{2}\left( x, y \right)}\cos^{2} \chi] \end{matrix} \right]$

(S6)

This Jones matrix $J \left( x, y \right)$ can perform a general transformation from orthogonal polarization states to independent complex amplitude profiles. As a validation, we use the calculated Jones matrix $J \left( x, y \right)$ of the metasurface to carry out transformation of an incident polarization state expressed as: $\left. |E_{in} \right\rangle{=[\begin{matrix} -sin \chi& e^{i\delta}\cos\chi\end{matrix} ]}^{T}$. This transformation is $\left. |E_{out} \right\rangle= J \left( x, y \right)\left. |E_{in} \right\rangle$ and shown as:

$$\left. |E_{out} \right\rangle=J \left( x, y \right)\left. |E_{in} \right\rangle=\left[ \begin{matrix} E_{1}\left( x, y \right)e^{{i\varphi}_{1}\left( x, y \right)}\cos^{2} \chi+E_{2}\left( x, y \right)e^{{i\varphi}_{2}\left( x, y \right)}\sin^{2} \chi& e^{-i\delta}\cos\chi\sin\chi\left[ E_{1}\left( x, y \right)e^{{i\varphi}_{1}\left( x, y \right)}-E_{2}\left( x, y \right)e^{{i\varphi}_{2}\left( x, y \right)} \right] \\ e^{-i\delta}\cos\chi\sin\chi[E_{1}\left( x, y \right)e^{{i\varphi}_{1}\left( x, y \right)}-E_{2}\left( x, y \right)e^{{i\varphi}_{2}\left( x, y \right)} & e^{-i2\delta}{[E}_{1}\left( x, y \right)e^{{i\varphi}_{1}\left( x, y \right)}\sin^{2} \chi+E_{2}\left( x, y \right)e^{{i\varphi}_{2}\left( x, y \right)}\cos^{2} \chi] \end{matrix} \right]\bullet\left[ \begin{matrix} -sin \chi\\ e^{i\delta}\cos\chi\end{matrix} \right]$$

$$=\left[ \begin{matrix} -E_{2}\left( x, y \right)e^{{i\varphi}_{2}\left( x, y \right)}( \sin^{3} \chi+\sin\chi\cos^{2} \chi) \\ E_{2}\left( x, y \right)e^{{i\varphi}_{2}\left( x, y \right)}e^{-i\delta}{(cos}^{3} \chi+\cos\chi\sin^{2} \chi) \end{matrix} \right]$$

$=E_{2}\left( x, y \right)e^{{i\varphi}_{2}\left( x, y \right)}\left[ \begin{matrix} - \sin\chi\\ e^{-i\delta}\cos\chi\end{matrix} \right]$ (S7)

It is clear from eq. S7 that the metasurface device can impart a complex amplitude profile $E_{2}\left( x, y \right)e^{{i\varphi}_{2}\left( x, y \right)}$ and the polarization of the output state ${[\begin{matrix} -sin \chi& e^{-i\delta}\cos\chi\end{matrix} ]}^{T}$ is the complex conjugate of the input polarization ${[\begin{matrix} -sin \chi& e^{i\delta}\cos\chi\end{matrix} ]}^{T}$. When changing this input polarization to its orthogonal polarization ${[\begin{matrix} \cos\chi& e^{i\delta}\sin\chi\end{matrix} ]}^{T}$, the corresponding mapping of Jones matrix $J \left( x, y \right)$ is identically suitable.

**Supplementary Note 2. The solutions for the required phase shifts and orientation angles of linear birefringent elements:**

As shown in the main text, the Jones matrix$J (x, y)$ for arbitrary orthogonal polarization pair is expressed as:

$J \left( x, y \right)=\left[ J_{A} \left( x, y \right)+J_{B} \left( x, y \right) \right]/2$ (S8)

where,

$J_{A} \left( x, y \right)=\left[ \begin{matrix} e^{{{i\varphi}_{A}}^{+}(x,y)}\cos^{2} \chi+e^{{{i\varphi}_{A}}^{-}(x,y)}\sin^{2} \chi& e^{-i\delta}\cos\chi\sin\chi[e^{{{i\varphi}_{A}}^{+}\left( x,y \right)}-e^{{{i\varphi}_{A}}^{-}\left( x,y \right)}] \\ e^{-i\delta}\cos\chi\sin\chi[e^{{{i\varphi}_{A}}^{+}\left( x,y \right)}-e^{{{i\varphi}_{A}}^{-}\left( x,y \right)}] & e^{-i2\delta}[e^{{{i\varphi}_{A}}^{+}\left( x,y \right)}\sin^{2} \chi+e^{{{i\varphi}_{A}}^{-}\left( x,y \right)}\cos^{2} \chi] \end{matrix} \right]$ (S9)

$J_{B} \left( x, y \right)=\left[ \begin{matrix} e^{{{i\varphi}_{B}}^{+}(x,y)}\cos^{2} \chi+e^{{{i\varphi}_{B}}^{-}(x,y)}\sin^{2} \chi& e^{-i\delta}\cos\chi\sin\chi[e^{{{i\varphi}_{B}}^{+}\left( x,y \right)}-e^{{{i\varphi}_{B}}^{-}\left( x,y \right)}] \\ e^{-i\delta}\cos\chi\sin\chi[e^{{{i\varphi}_{B}}^{+}\left( x,y \right)}-e^{{{i\varphi}_{B}}^{-}\left( x,y \right)}] & e^{-i2\delta}[e^{{{i\varphi}_{B}}^{+}\left( x,y \right)}\sin^{2} \chi+e^{{{i\varphi}_{B}}^{-}\left( x,y \right)}\cos^{2} \chi] \end{matrix} \right]$ (S10)

and

$${\varphi_{A}}^{+}\left( x,y \right)=\cos^{-1}[ E_{1}\left( x, y \right)]+\varphi_{1}\left( x, y \right)$$

$${\varphi_{A}}^{-}\left( x,y \right)=\cos^{-1} [E_{2}\left( x, y \right)]+\varphi_{2}\left( x, y \right)$$

$${\varphi_{B}}^{+}\left( x,y \right)=\varphi_{1}\left( x, y \right)-\cos^{-1} [E_{1}\left( x, y \right)]$$

$${\varphi_{B}}^{-}\left( x,y \right)=\varphi_{2}\left( x, y \right)-\cos^{-1} [E_{2}\left( x, y \right)]$$

For the most general case of elliptical polarization, although the eigenvalues and eigenvectors of $J_{A} \left( x, y \right)$ and $J_{B} \left( x, y \right)$ do not yield clear analytical solutions for the required phase shifts and orientation angles of nanopillars A and B, the numerical solutions can still be calculated for special values of $E_{1,2}\left( x, y \right)$, $\varphi_{1,2}\left( x, y \right)$, $\chi$ and $\delta$.

In this work, we chose two typical cases of orthogonal circular and linear polarizations to demonstrate the ability of the proposed metasurface platform to manipulate the wavefront of light. Firstly, for the circular polarization, $\chi=\pi/4$ and $\delta=\pi/2$, in terms of unitary matrix, eq. S9 and eq. S10 are simplified as:

$J_{A} \left( x, y \right)=\frac{1}{2}\left[ \begin{matrix} e^{{{i\varphi}_{A}}^{+}(x,y)}+e^{{{i\varphi}_{A}}^{-}(x,y)} & -ie^{{{i\varphi}_{A}}^{+}(x,y)}+ie^{{{i\varphi}_{A}}^{-}(x,y)} \\ -ie^{{{i\varphi}_{A}}^{+}(x,y)}+ie^{{{i\varphi}_{A}}^{-}(x,y)} & -e^{{{i\varphi}_{A}}^{+}\left( x,y \right)}-e^{{{i\varphi}_{A}}^{-}(x,y)} \end{matrix} \right]$ (S11)

$J_{B} \left( x, y \right)=\frac{1}{2}\left[ \begin{matrix} e^{{{i\varphi}_{B}}^{+}(x,y)}+e^{{{i\varphi}_{B}}^{-}(x,y)} & -ie^{{{i\varphi}_{B}}^{+}(x,y)}+ie^{{{i\varphi}_{B}}^{-}(x,y)} \\ -ie^{{{i\varphi}_{B}}^{+}(x,y)}+ie^{{{i\varphi}_{B}}^{-}(x,y)} & -e^{{{i\varphi}_{B}}^{+}\left( x,y \right)}-e^{{i\varphi_{B}}^{-}(x,y)} \end{matrix} \right]$ (S12)

Due to symmetric and unitary conditions, by solving the characteristic equations of $J_{A} \left( x, y \right)$ and $J_{B} \left( x, y \right)$, their corresponding eigenvalues can be calculated as:

For $J_{A} \left( x, y \right):$ $\xi_{A1}=e^{i\frac{1}{2}[{\varphi_{A}}^{+}\left( x,y \right)+{\varphi_{A}}^{-}\left( x,y \right)]}$ $\xi_{A2}=e^{i\frac{1}{2}[{\varphi_{A}}^{+}\left( x,y \right)+{\varphi_{A}}^{-}\left( x,y \right)]-i\pi}$ (S13)

For $J_{B} \left( x, y \right):$ $\xi_{B1}=e^{i\frac{1}{2}[{\varphi_{B}}^{+}\left( x,y \right)+{\varphi_{B}}^{-}\left( x,y \right)]}$ $\xi_{B2}=e^{i\frac{1}{2}[{\varphi_{B}}^{+}\left( x,y \right)+{\varphi_{B}}^{-}\left( x,y \right)]-i\pi}$ (S14)

and eigenvectors are calculated as:

For $J_{A} \left( x, y \right)$: $V_{A1} = \left[ \begin{matrix} \cos\theta_{A} & \sin\theta_{A} \end{matrix} \right]^{T}$ ${V_{A2} =\left[ -\begin{matrix} \sin\theta_{A} & \cos\theta_{A} \end{matrix} \right]}^{T}$ (S15)

For $J_{B} \left( x, y \right)$: $V_{B1} = \left[ \begin{matrix} \cos\theta_{B} & \sin\theta_{B} \end{matrix} \right]^{T}$ ${V_{B2} =\left[ -\begin{matrix} \sin\theta_{B} & \cos\theta_{B} \end{matrix} \right]}^{T}$ (S16)

where, $\theta_{A}=\frac{1}{4}[{\varphi_{A}}^{+}\left( x,y \right)+{\varphi_{A}}^{-}\left( x,y \right)]$, $\theta_{B}=\frac{1}{4}[{\varphi_{B}}^{+}\left( x,y \right)+{\varphi_{B}}^{-}\left( x,y \right)]$. Thus, the Jones matrix $J (x, y)$ can be rewritten in terms of eigenvectors and eigenvalue of $J_{A} \left( x, y \right)$ and $J_{B} \left( x, y \right)$ as:

$$J\left( x, y \right)=\left[ R_{A}\Lambda_{A}{R_{A}}^{-1}+R_{B}\Lambda_{B}{R_{B}}^{-1} \right]/2$$

$=\frac{1}{2}\left[ \begin{matrix} V_{A1} & V_{A2} \end{matrix} \right]\left[ \begin{matrix} \xi_{A1} & 0 \\ 0 & \xi_{A2} \end{matrix} \right]\left[ \begin{matrix} V_{A1} & V_{A2} \end{matrix} \right]^{-1}+\frac{1}{2}\left[ \begin{matrix} V_{B1} & V_{B2} \end{matrix} \right]\left[ \begin{matrix} \xi_{B1} & 0 \\ 0 & \xi_{B2} \end{matrix} \right]\left[ \begin{matrix} V_{B1} & V_{B2} \end{matrix} \right]^{-1}$ (S17)

Since the matrix $J_{A} \left( x, y \right)$ and $J_{B} \left( x, y \right)$ operates in the linear polarization basis, the diagonal matrix $\Lambda_{A}$ and $\Lambda_{B}$ determines the phase shifts $\delta_{Ax}=\frac{1}{2}[{\varphi_{A}}^{+}\left( x,y \right)+{\varphi_{A}}^{-}\left( x,y \right)]$ and $\delta_{Ay}=\frac{1}{2}[{\varphi_{A}}^{+}\left( x,y \right)+{\varphi_{A}}^{-}\left( x,y \right)]-\pi$ along the two symmetry axes of the linearly birefringent element A and $\delta_{Bx}=\frac{1}{2}[{\varphi_{B}}^{+}\left( x,y \right)+{\varphi_{B}}^{-}\left( x,y \right)]$ and $\delta_{By}=\frac{1}{2}[{\varphi_{B}}^{+}\left( x,y \right)+{\varphi_{B}}^{-}\left( x,y \right)]-\pi$ along the two symmetry axes of the linearly birefringent element B, respectively. Meanwhile, the matrix $R_{A}$ and $R_{B}$ correspond to the rotation matrix for $\Lambda_{A}$ and $\Lambda_{B}$ and determine the rotation angle $\theta_{A} =\frac{1}{4}[{\varphi_{A}}^{+}\left( x,y \right)-{\varphi_{A}}^{-}\left( x,y \right)]$ and $\theta_{B} =\frac{1}{4}[{\varphi_{B}}^{+}\left( x,y \right)-{\varphi_{B}}^{-}\left( x,y \right)]$ of the fast axis of birefringent elements A and B in the *x*-*y* plane, respectively.

Secondly, for orthogonal linear polarizations, $\chi=0$ and $\delta=0$, Jones matrix $J \left( x, y \right)$ is simplified as:

$$J \left( x, y \right)=\frac{1}{2}\left[ \begin{matrix} e^{{{i\varphi}_{A}}^{+}(x,y)}+e^{{{i\varphi}_{B}}^{+}(x,y)} & 0 \\ 0 & e^{{{i\varphi}_{A}}^{-}\left( x,y \right)}+e^{{{i\varphi}_{B}}^{-}(x,y)} \end{matrix} \right]$$

$={[J}_{A} \left( x, y \right)+J_{B} \left( x, y \right)]/2$

$=\frac{1}{2}\left[ \begin{matrix} e^{{{i\varphi}_{A}}^{+}(x,y)} & 0 \\ 0 & e^{{{i\varphi}_{A}}^{-}\left( x,y \right)} \end{matrix} \right]+\frac{1}{2}\left[ \begin{matrix} e^{{{i\varphi}_{B}}^{+}(x,y)} & 0 \\ 0 & e^{{{i\varphi}_{B}}^{-}(x,y)} \end{matrix} \right]$ (S18)

According to the diagonal matrices of $J_{A} \left( x, y \right)$ and $J_{B} \left( x, y \right)$, we can directly obtain the phase shifts $\delta_{Ax}={\varphi_{A}}^{+}(x,y)$ and $\delta_{Ay}={\varphi_{A}}^{-}(x,y)$ along the symmetry axes of linearly birefringent nanopillar A and $\delta_{Bx}={\varphi_{B}}^{+}(x,y)$ and $\delta_{By}={\varphi_{B}}^{-}(x,y)$ along the symmetry axes of linearly birefringent nanopillar B, respectively. Note that no rotation of the nanopillars are needed.

In summary, in accordance with requirements and given arbitrary amplitude and phase profiles, we can use the above formulas to design metasurface devices for complete and independent amplitude and phase control of orthogonal states of polarization. In order to implement $J \left( x, y \right)$, a set of subwavelength nanopillars are designed to provide the required phase shifts and orientation angles at any point (*x*, *y*) of the metasurface.


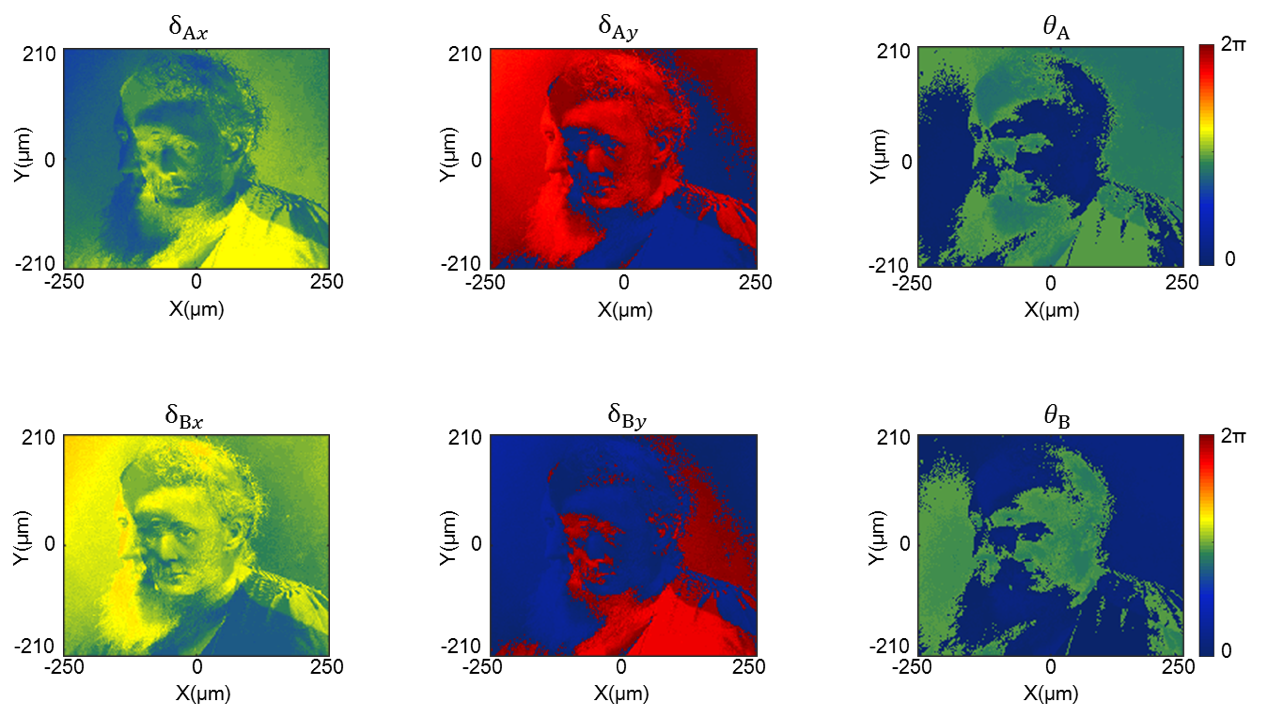


**Fig. S1.** The phase shifts ${(\delta}_{Ax},\delta_{Ay},\delta_{Bx},\delta_{By})$ and rotation angle $(\theta_{A},\theta_{B})$ of nanopillars A and B as a function of the spatial coordinates in the plane of metasurface MF1 for chirality-switchable nanoprinting (Kelvin’s portrait for RCP and Madame Curie’s portrait for LCP).


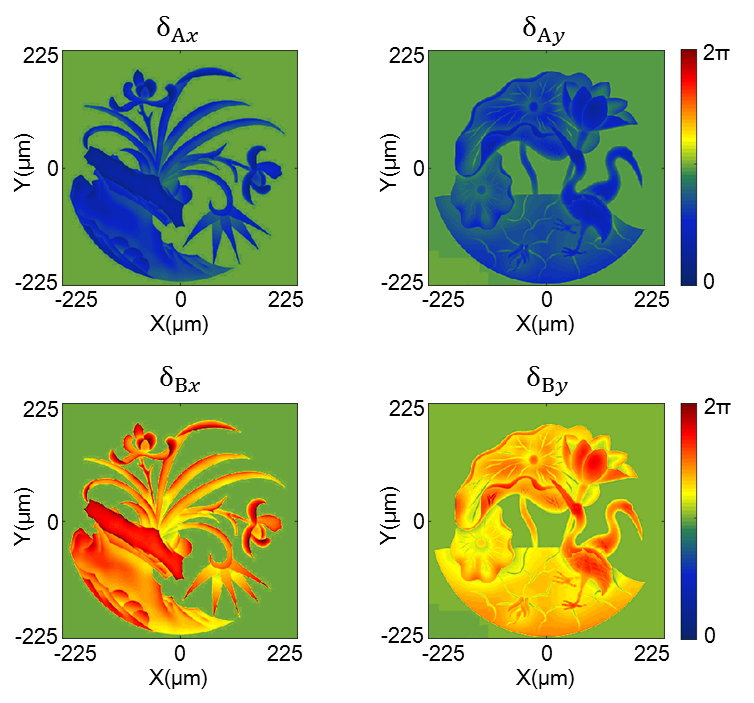


**Fig. S2.** The phase shifts ${(\delta}_{Ax},\delta_{Ay},\delta_{Bx},\delta_{By})$ of nanopillars A and B as a function of the spatial coordinates in the plane of metasurface MF2 for linear polarization-switchable nanoprinting (“orchids” for *x*-polarized light and “lotuses” for *y*-polarized light).

**
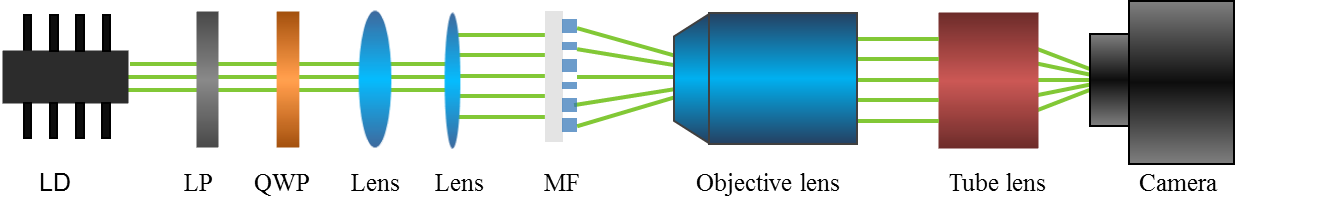
**

**Fig. S3.** Experimental setup for measurement of fabricated metasurface devices. A semiconductor laser passing through a linear polarizer (LP) and quarter waveplate (QWP) are converted into the desired polarization lights and incident on the metasurfaces. The light is collected by a 20× objective lens imaged on a charge coupled device (CCD) camera through a tube lens. For orthogonal linear polarization measurements, the QWP is replaced by a half waveplate (HWP).


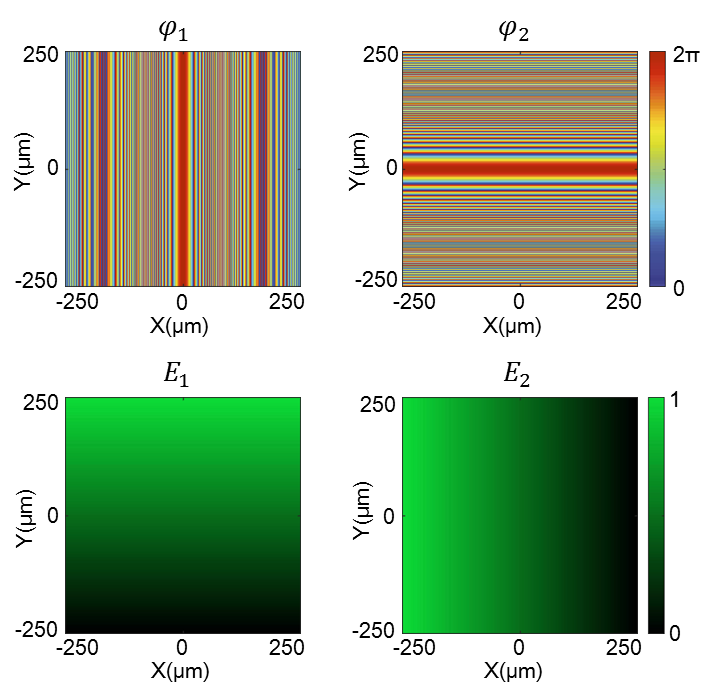


**Fig. S4**. The cylindrical lens phase profiles ($\varphi_{1,} \varphi_{2}$) and custom-design amplitude profiles ($E_{1,} E_{2})$ corresponding to two orthogonal circular polarization encoded on the metasurface MF3 for chirality-switchable cylindrical lens focusing with non-uniform intensity distributions.


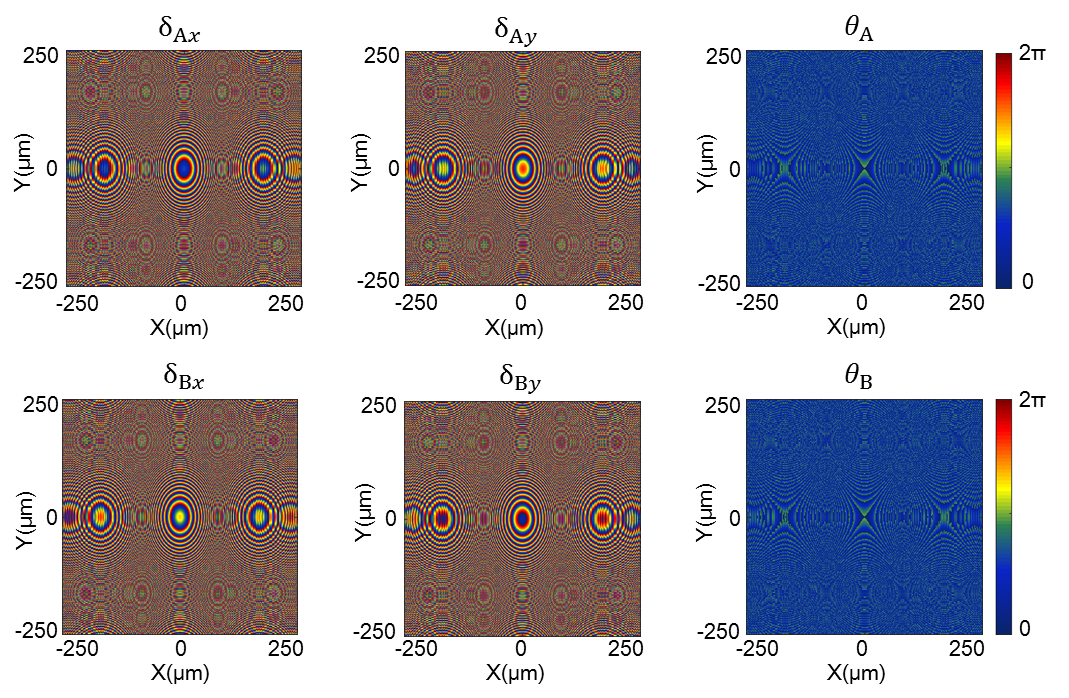


**Fig. S5**. The phase shifts ${(\delta}_{Ax},\delta_{Ay},\delta_{Bx},\delta_{By})$ and rotation angle $(\theta_{A},\theta_{B})$ of nanopillars A and B as a function of the spatial coordinates in the plane of metasurface MF3 for chirality-switchable cylindrical lens focusing with non-uniform intensity distributions.


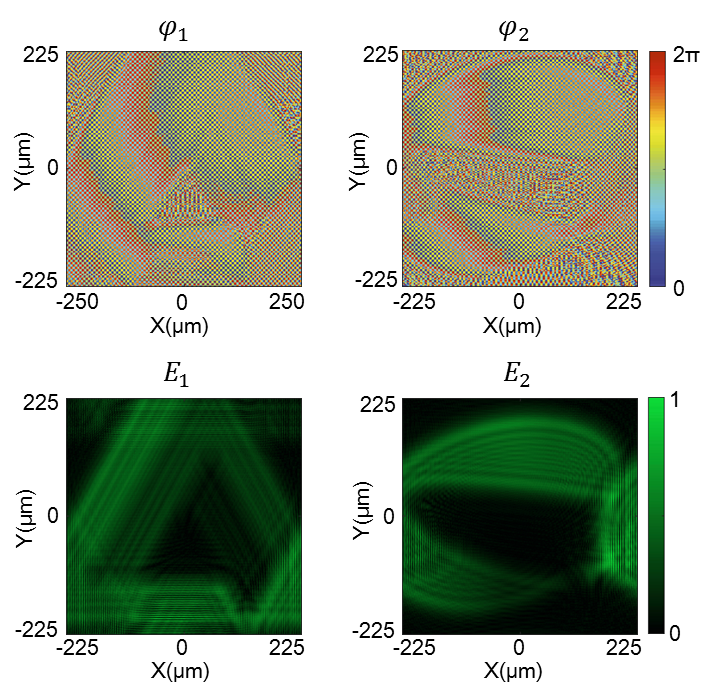


**Fig. S6**. The near-field phase ($\varphi_{1,}\varphi_{2}$) and amplitude ($E_{1,}E_{2})$ profiles corresponding to two orthogonal linear polarization encoded on the metasurface MF4 for polarization-switchable complex amplitude hologram (‘Penrose triangle’ for *x*-polarized light and ‘Mobius strip’ for *y*-polarized light).


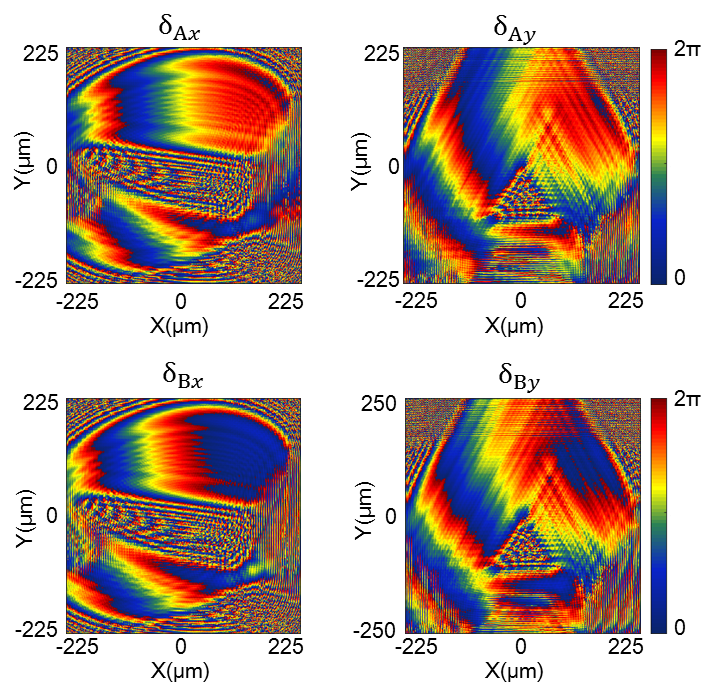


**Fig. S7**. The phase shifts ${(\delta}_{Ax},\delta_{Ay},\delta_{Bx},\delta_{By})$ of nanopillars A and B as a function of the spatial coordinates in the plane of metasurface MF4 for polarization-switchable complex amplitude hologram.


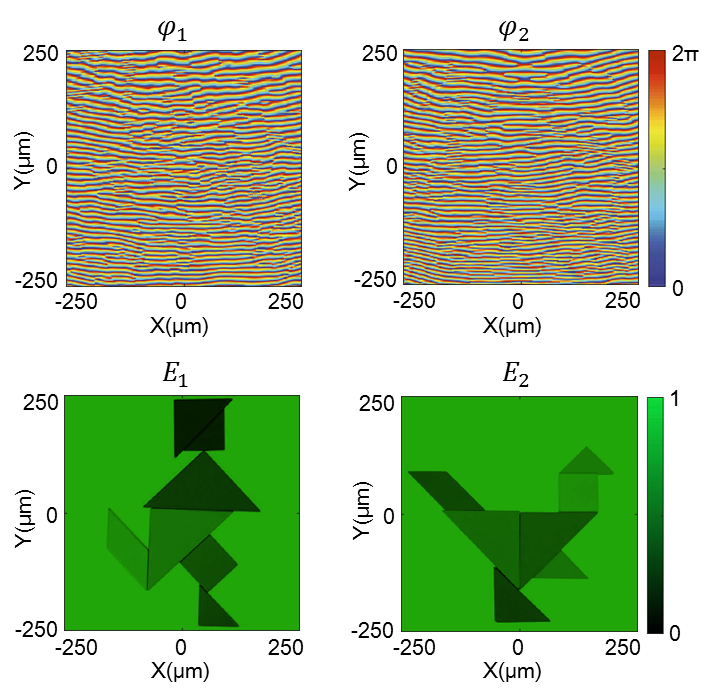


**Fig. S8**. The near-field phase ($\varphi_{1,}\varphi_{2}$) and amplitude ($A_{1,}A_{2})$ profiles corresponding to two orthogonal circular polarization encoded on the metasurface MF5 for synchronous generation of chirality-switchable nanoprinting-hologram images.


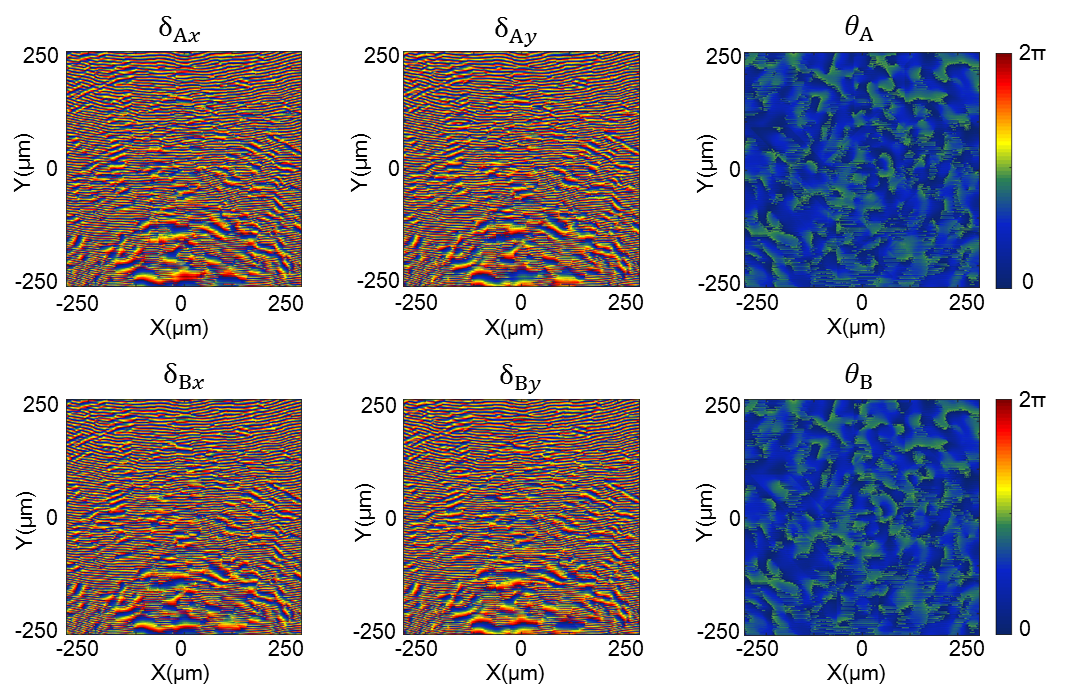


**Fig. S9**. The phase shifts ${(\delta}_{Ax},\delta_{Ay},\delta_{Bx},\delta_{By})$ and rotation angle $(\theta_{A},\theta_{B})$ of nanopillars A and B as a function of the spatial coordinates in the plane of metasurface MF5.


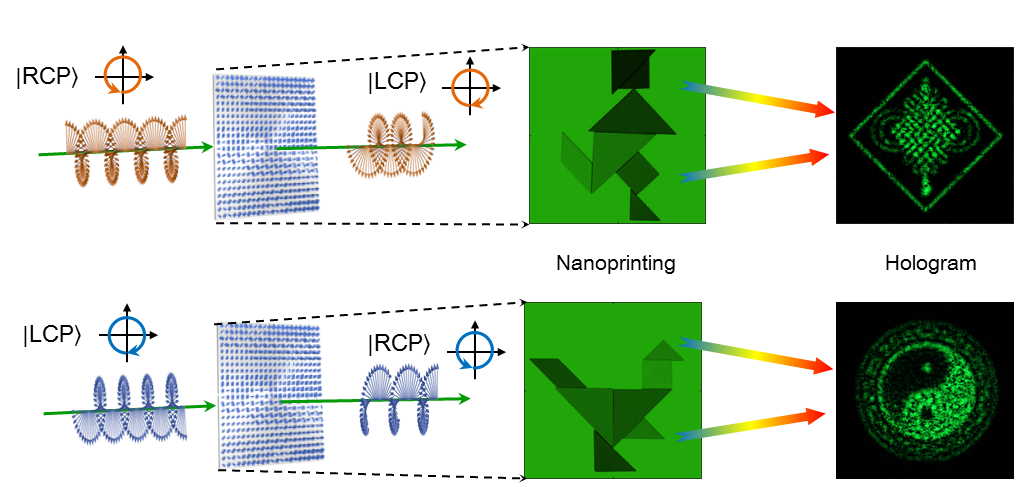


**Fig. S10. Simulated hologram images for RCP and LCP light**. According to the computed phase ($\varphi_{1,}\varphi_{2}$) and target amplitude ($A_{1,}A_{2}$) profiles of the metasurface MF5, the reconstructed hologram images for RCP and LCP incident light are simulated in the far field.


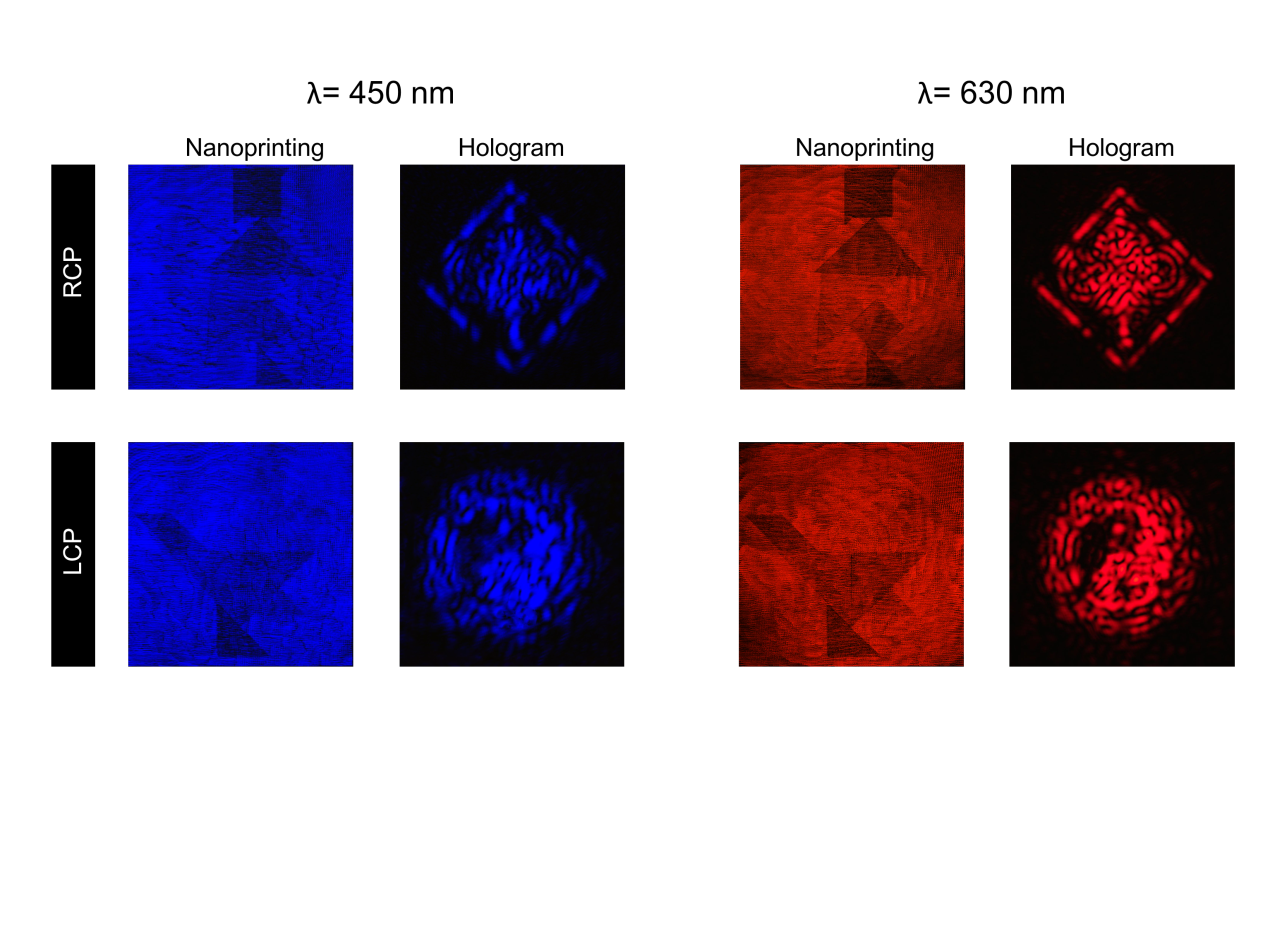


**Fig. S11.** Broadband response of the fabricated metasurface MF5 for four-channel nanoprinting-hologram. The nanoprinting and holographic images are measured at wavelengths of 450 nm (blue) and 630 nm (red), respectively.


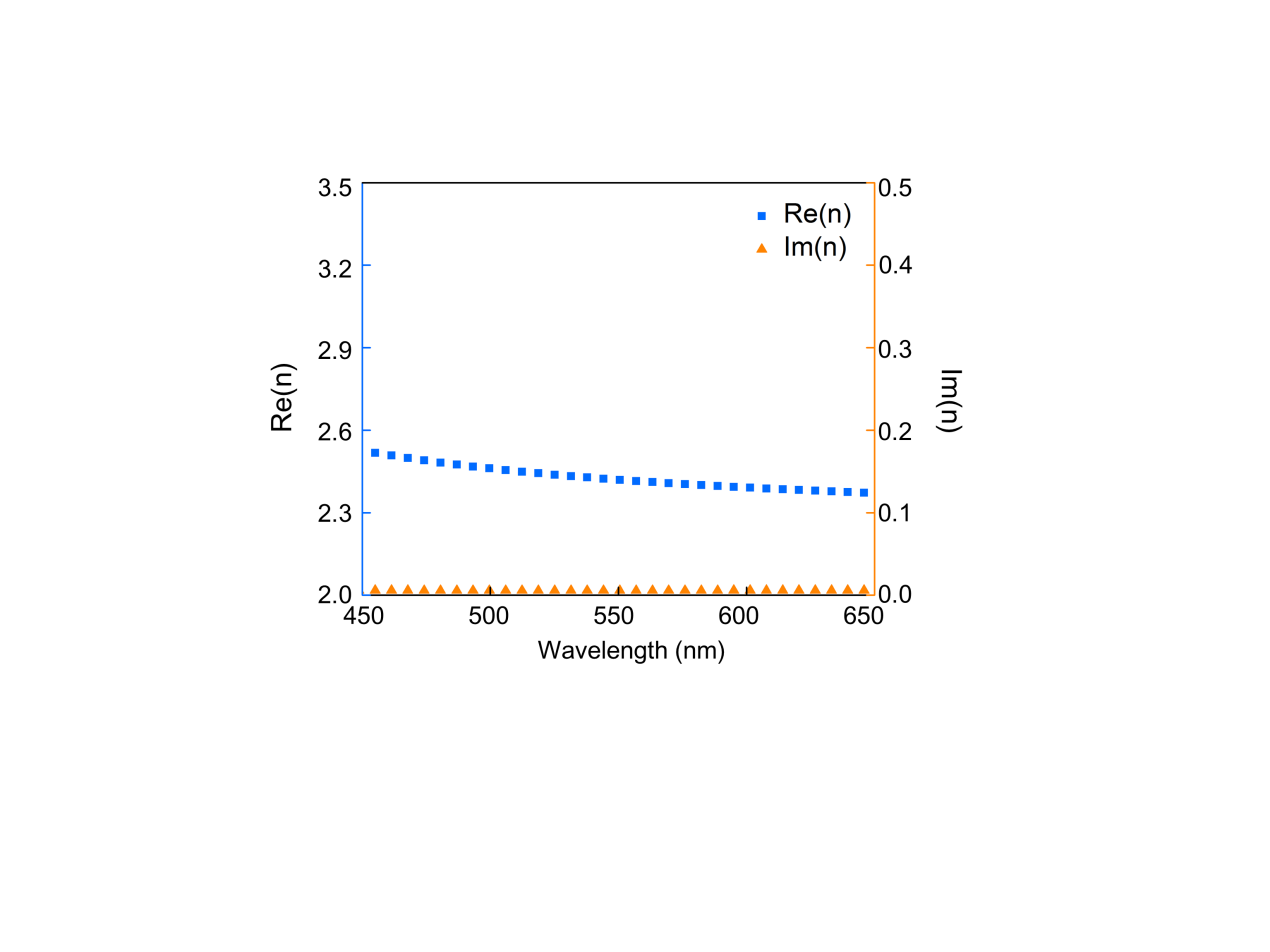


**Fig. S12.** Complex refractive index of atomic layer deposition (ALD) amorphous titanium dioxide (TiO_2_). The real (blue squares) and imaginary (orange triangles) part of the complex refractive index as a function of wavelength are measured by spectroscopic ellipsometry.


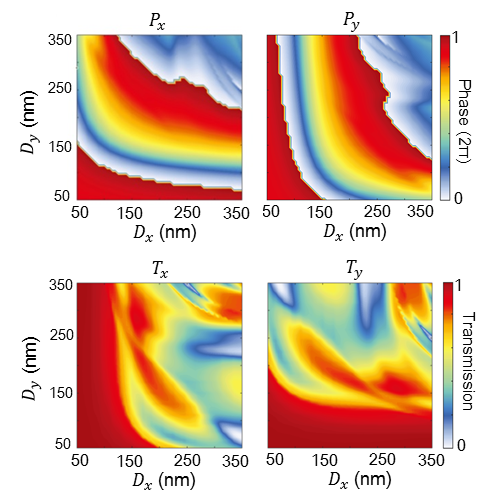


**Fig. S13.** Simulated phase shifts (*P_x_* and *P_y_*) and power transmission coefficients (*T_x_* and *T_y_*) for *x*- and *y*- polarized light as functions of the rectangular nanopillar diameters ($D_{x}$ and $D_{y}$) at a free-space wavelength of 530 nm.


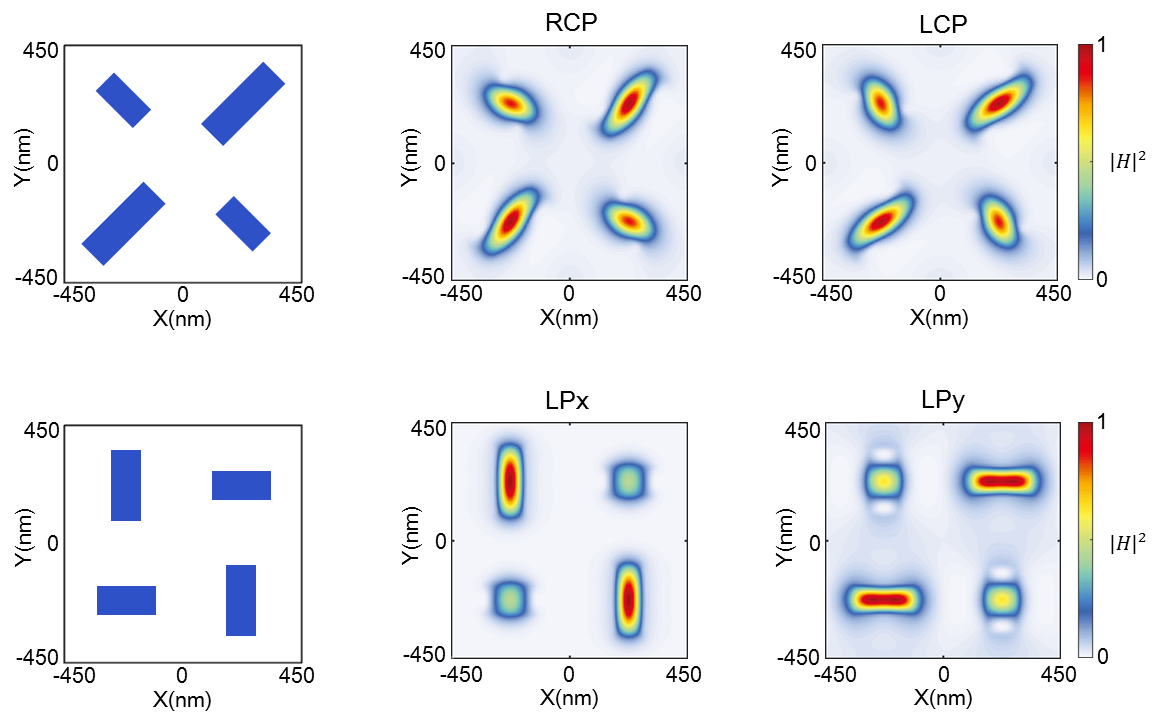


**Fig. S14.** Simulated normalized magnetic-field energy density of the nanopillar array at an incident wavelength of 530 nm. First panel: A metasurface super-pixel composed of nanopillar A with $D_{Ax}=200 nm$, $D_{Ay}=100 nm$ and $\theta_{A}=3\pi/4$ and nanopillar B with $D_{Bx}=335 nm$, $D_{By}=120 nm$ and $\theta_{B}=\pi/4$. The normalized magnetic-field energy density is simulated when RCP and LCP light is incident on the metasurface super-pixel. Second panel: A metasurface super-pixel composed of nanopillar A with $D_{Ax}=115 nm$, $D_{Ay}=270 nm$ and $\theta_{A}=0$ and nanopillar B with $D_{Bx}=225 nm$, $D_{By}=110 nm$ and $\theta_{B}=0$. The normalized magnetic-field energy density is simulated when *x*- and *y*-polarized light is incident on the metasurface super-pixel.
